# Clearing method for 3-dimensional immunofluorescence of osteoarthritic subchondral human bone reveals peripheral cholinergic nerves

**DOI:** 10.1038/s41598-020-65873-6

**Published:** 2020-06-01

**Authors:** Alice Courties, Morgane Belle, Simge Senay, Adeline Cambon-Binder, Alain Sautet, Alain Chédotal, Francis Berenbaum, Jérémie Sellam

**Affiliations:** 1 Sorbonne Université, Paris, France; 2 INSERM UMRS_938, CRSA, Paris, France; 3 Department of Rheumatology, Assistance Publique - Hôpitaux de Paris (AP-HP), Saint-Antoine Hospital, Paris, France; 4 INSERM, CNRS, Institut de la Vision, Paris, France; 5 Department of Orthopedic Surgery, AP-HP, Saint-Antoine Hospital, Paris, France

**Keywords:** Bone, Neurophysiology

## Abstract

The cholinergic system plays a major anti-inflammatory role in many diseases through acetylcholine (Ach) release after vagus nerve stimulation. Osteoarthritis (OA) is associated with local low-grade inflammation, but the regulatory mechanisms are unclear. Local Ach release could have anti-inflammatory activity since articular cells express Ach receptors involved in inflammatory responses. Using the 3DISCO clearing protocol that allows whole-sample 3-dimensional (3D) analysis, we cleared human OA cartilage-subchondral bone samples to search for cholinergic nerve fibres able to produce Ach locally. We analysed 3 plugs of knee cartilage and subchondral bone from 3 OA patients undergoing arthroplasty. We found no nerves in the superficial and intermediate articular cartilage layers, as evidenced by the lack of Peripherin staining (a peripheral nerves marker). Conversely, peripheral nerves were found in the deepest layer of cartilage and in subchondral bone. Some nerves in the subchondral bone samples were cholinergic because they coexpressed peripherin and choline acetyltransferase (ChAT), a specific marker of cholinergic nerves. However, no cholinergic nerves were found in the cartilage layers. It is therefore feasible to clear human bone to perform 3D immunofluorescence. Human OA subchondral bone is innervated by cholinergic fibres, which may regulate local inflammation through local Ach release.

## Introduction

Osteoarthritis (OA) is the most common joint disease and is a considerable burden on quality of life due to joint pain, stiffness and loss of function^1^. OA is mostly characterized by cartilage degradation but is now considered a whole-joint disease associated with subchondral bone remodelling and synovitis. Articular cartilage is composed of several layers, and the deepest layer, called calcified cartilage, is located on top of subchondral bone. Articular cartilage is a unique tissue that is not innervated or vascularized. Communication between subchondral bone and calcified cartilage is needed for cartilage homeostasis. Such communication is also involved in the OA process because of the circulation of soluble mediators from subchondral bone to cartilage undergoing alterations^2^. Interplay between subchondral bone and deep cartilage may involve micro-cracks at the junction between the two tissues as well as neoangiogenesis of the calcified cartilage, which could be directly involved in OA pathophysiology^3–5^. Additionally, neurogenesis may play a role in OA pathophysiology. Walsh and colleagues previously showed that during OA, peripheral sensory and sympathetic nerves grow in calcified cartilage and may be involved in OA pain^6,7^.

Beyond sensory mediators and sensory nerves, the parasympathetic system has been reported to have anti-inflammatory properties through the action of its mediator, acetylcholine (Ach), on nicotinic Ach receptors and, in particular, the alpha7 nicotinic receptor^8,9^. Ach production is controlled by choline acetyltransferase (ChAT), which is mainly recognized as a marker of cholinergic nerves. Because Ach interacts locally with nicotinic Ach and muscarinic receptors expressed by resident cells of the bone to exert anti-inflammatory effects, we wondered whether cholinergic nerves are locally expressed in subchondral bone^10,11^. Because it is challenging to characterize nerve structures using two-dimensional (2D) techniques with confocal microscopy, we adapted a recently published protocol for the three-dimensional (3D) analysis of immunofluorescence^12,13^. 3D immunolabelling allows the study of anatomical structures while preserving the whole structure of the tissue sample. This method was first developed in neuroscience for studying the mouse brain and spinal cord and then for studying embryonic development in humans^13^. Improvements in light sheet microscopy and the clearing protocol have made it possible to readily immunolabel a whole tissue sample in a straightforward manner. Therefore, this technique is of interest for the study of small structures, such as nerves. One challenging step is clearing bone because it is rich in adipose tissue and because decalcification techniques complicate the protocol and may limit its success. Several teams have managed to clear murine bone in different stages of differentiation, but until now, no one has managed to clear human adult bone^14,15^. The objective of our study was to determine the presence of cholinergic nerves in cartilage and subchondral OA human samples using 3D immunolabelling.

## Results

### The 3DISCO clearing protocol enabled human OA subchondral bone immunofluorescence

**Figure 1:**
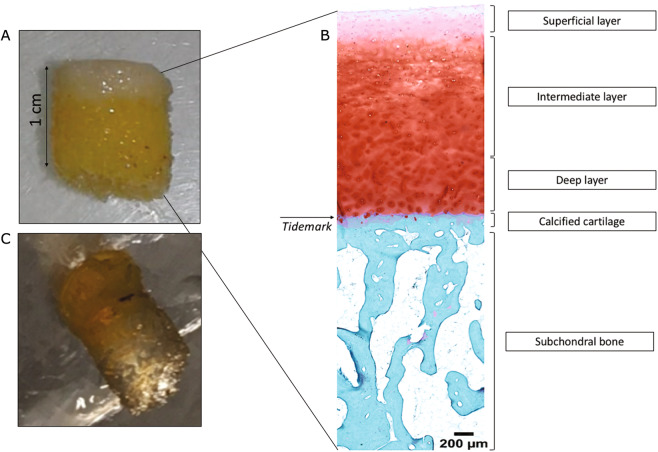
Human OA plug analysis. (**A**) Osteoarthritic human subchondral bone/cartilage plugs obtained from a macroscopically healthy zone of an osteoarthritic knee during arthroplasty for knee OA. (**B**) Histological analysis of a human subchondral bone/cartilage plug after paraformaldehyde fixation, decalcification, paraffin embedding and Safranin O/Fast Green staining for the analysis of all cartilage layers and subchondral bone. (**C**) Osteoarthritic human subchondral bone/cartilage plug after the 3DISCO clearing protocol.

**Figure 2:**
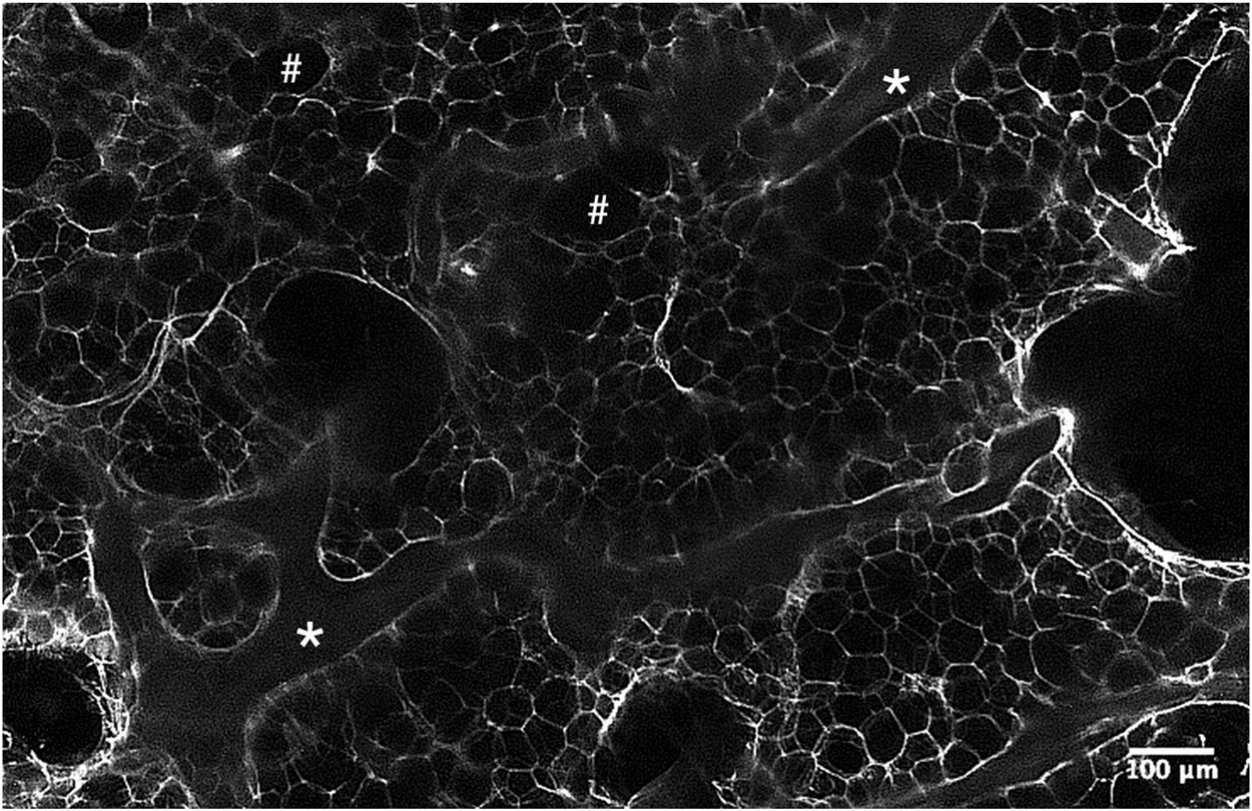
Light-sheet microscopy analysis of human OA subchondral bone after the clearing protocol. The samples analysed is a cleared subchondral plug, stained with peripherin. This is a zone without nerves in a higher magnification to precisely look to the structure: * for trabecular bone and # for bone marrow area.

**Figure 3:**
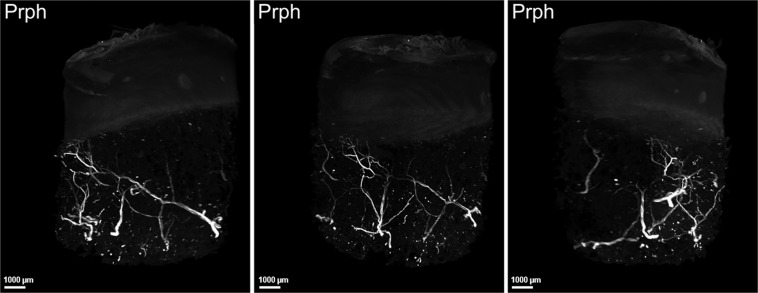
Human OA subchondral bone has peripheral nerves that invade deep layer of cartilage. 3-dimensional analysis of peripheral nerves in human OA subchondral bone using peripherin (Prph) labelling after the 3DISCO clearing protocol (see video 1). The three pictures show the same sample in 3 different views. Peripheral nerve in the deep cartilage layer coming from subchondral bone as shown by peripherin labelling (white), Prph: peripherin.

We first used one sample to establish that plugs of a particular size could be analysed by microscopy, and we confirmed that all cartilage layers and sufficient subchondral bone were present for analysis (Fig. 1A,B). Then, we performed immunofluorescence on three independent samples from patients aged 68 to 79 years, 1 woman and two men, with a body mass index between 32 and 38 kg/m^2^. We first confirmed that the clearing method of cartilage-subchondral bone samples worked using the 3DISCO protocol. We found that the 3DISCO protocol allowed us to macroscopically clear all subchondral bone (Fig. 1C) and to precisely analyse the anatomical structure of trabecular bone, allowing us to differentiate trabecular bone and bone marrow, as shown in Fig. 2. We confirmed that immunofluorescence was an efficient technique for localizing peripheral nerves in the 3 samples using peripherin labelling (Fig. 3, Supplementary video 1). The nerve density appeared to be similar in the 3 samples. All the nerves appeared to come from the epiphyseal region of the bone and cross all the subchondral bone to reach the cartilage. Indeed, as expected, no nerves were present in superficial and the intermediate cartilage, although some peripherin-positive nerve fibres had invaded deeper layers of cartilage in the 3 samples (Fig. 3, Supplementary video 1).

### Cholinergic peripheral nerves are present in human OA subchondral bone

**Figure 4:**
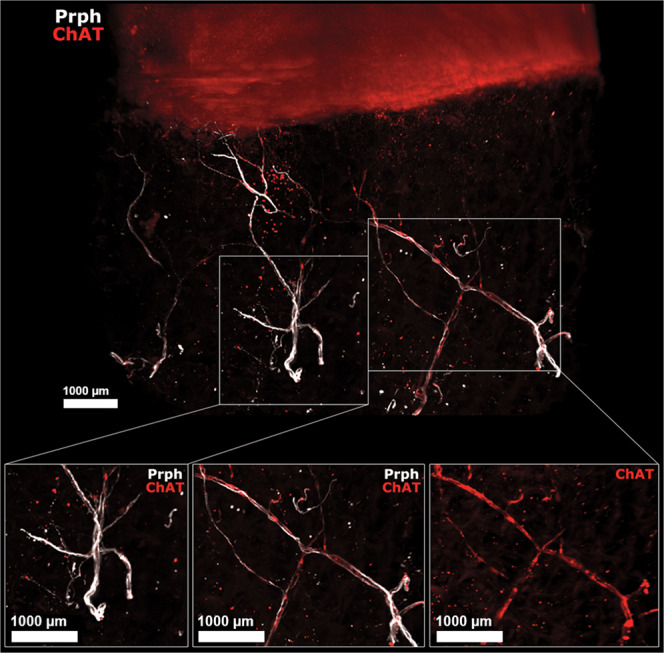
Human OA subchondral bone has peripheral and cholinergic nerves. Immunofluorescence of peripherin and ChAT in human OA subchondral bone (see video 2) after the 3DISCO clearing protocol and analysis with Imaris. The colocalization of both markers showed that some subchondral bone peripheral nerves marked by Prph (white) are also cholinergic, as they showed ChAT immunofluorescence (red) as shown on the right while some did not expressed ChAT (left). ChAT: choline acetyltransferase, Prph: peripherin.

In the three independent human OA samples, some of the peripheral nerves coexpressed ChAT, meaning that some of the fibres may produce ACh and thus belong to the cholinergic nervous system (Fig. 4, Supplementary video 2). However, we did not find any cholinergic nerves in any of the cartilage layers. No nerves structures or cholinergic nerves was shown in the two negative controls that we performed (one without any antibody and one with the secondary antibody at the same concentration) as shown in the Supplementary Files 3.

## Discussion

We report for the first time that cholinergic fibres are present in human OA subchondral bone. Furthermore, we show that a clearing protocol for cartilage-subchondral bone samples is feasible and enables 3D immunolabelling of these human tissues. This study opens new avenues of research in bone analysis and will allow the qualitative and quantitative analysis of anatomical structures in human diseases.

Parasympathetic innervation of joint tissues has been less studied than its sympathetic counterpart. Bajayo *et al*. successfully demonstrated parasympathetic innervation of rat bone by injecting a labelled neurogenic pseudorabies virus into the metaphyseal region of a rat femur to label parasympathetic sacral centres^16^. However, these researchers did not confirm their results in human samples. Others have reported the presence of cholinergic fibres in the periosteum of rats based on expression of vesicular Ach transporter (VAchT) or ChAT^17^. Cholinergic fibres have also been found in periarticular tissues (mostly skin and muscle) from mice with arthritis, which have decreased sympathetic fibre density. VAchT-positive fibres were also found in very few synovia of OA and rheumatoid arthritis patients^18^. The presence of cholinergic fibres does not necessarily indicate the presence of a complete parasympathetic system, as these fibres could arise from sympathetic fibres by modifying their phenotype. In the periosteum, cholinergic fibres are derived from catecholaminergic fibres in response to local neuropoietic cytokines, such as leukaemia inhibitory factor, which is produced locally by osteoclasts or osteoblasts^17–20^. These apparent cholinergic fibres in subchondral bone could arise from sympathetic rather than parasympathetic centres. Despite the final anatomical provenance of the nerve, the presence of cholinergic nerves indicates the local production of Ach, which can be modulated and can act on nicotinic and/or muscarinic cholinergic receptors to exert anti-inflammatory and immunomodulatory activity or to modulate bone mass, which is of interest for future research in osteoporosis^10,21,22^. However, this study did not allow to quantify these cholinergic nerves and to correlate such a quantification with OA severity and our results involve only healthy macroscopically zone of human OA subchondral bone. Furthermore, we cleared and immunolabelled 3D human subchondral bone for the first time. Clearing some tissues remains a challenge and constitutes a limiting step for 3D immunolabelling. The principle of clearing is to modify the refractive index of the tissue by changing its composition; this is accomplished by removing the lipids and then immersing the samples in a clearing medium to achieve a uniform refractive index. Several clearing protocols have been published in the last decade^23^; the most commonly used protocols are the 3DISCO and iDISCO protocols, which both employ the same optical clearing approach and a dibenzyl ether-based technique^24,25^. Not all antibodies are compatible with clearing protocol. However, we have used two antibodies already used with this method and published^13^. Thus, this antibody of ChAT stained motoneurons on human embryos (https://transparent-human-embryo.com/?p=863). However, although filtered, the immunostaining of ChAT was slightly alterated with the presence of non-specific labelling. Since these artefacts were not found when the sample was incubated with the secondary antibody alone, it is likely that they were due to the primary antibody. Another explanation could have been the expression of a non-neuronal cholinergic system by non-neuronal cells in the bone but it would have led to much more staining in the entire sample.

We demonstrated that the 3DISCO protocol is feasible and can be used for future research on nerve and vessels in joint tissues as well as for cell marker labelling.

In conclusion, cholinergic fibres are found in the macroscopically healthy area of the human OA subchondral bone as to deliver locally Ach. The 3D clearing protocol developed for human cartilage and bone allows the analysis of whole cartilage-bone samples of 10 mm^2^ without sectioning.

## Methods

Four independent human OA samples were isolated from the knees of patients undergoing joint arthroplasty for OA at AP-HP Saint-Antoine Hospital (Paris, France). Informed consent for the use of tissue was obtained from each patient before surgery. Experiments using human samples were approved by a French Institutional Review Board (Comité de Protection des Personnes, Paris Ile de France 5 and Commission Nationale de l’Informatique et des Libertés). All experiments were performed in accordance with relevant guidelines and regulations. Just after the surgery, small 10-mm^2^ plugs of human OA cartilage and subchondral were obtained. Each plug was taken from a macroscopically healthy area of the joint and included the entire cartilage layer and a piece of subchondral bone just below the cartilage. Samples were fixed at 4 °C for 24 hours in 4% paraformaldehyde diluted in PBS and then decalcified in 500 mM EDTA (pH 7.4, VWR, Pennsylvania, USA) diluted in PBS for approximately 4 weeks at 4 °C in a rotating wheel. The EDTA solution was changed every 48 hours until the samples were considered decalcified (approximately 4 weeks). One sample was used for standard histological analysis and embedded in paraffin. Sagittal sections of 5 µm were obtained, and one slide was stained with Safranin O/Fast Green for the analysis of cartilage layers and subchondral bone structure. For 3D immunofluorescence, the three other samples were rinsed three times in PBS before being dehydrated in a series of methanol/PBS (50% methanol/PBS, 80% methanol/PBS and 100% methanol) at room temperature for 1.5 hours. The samples were incubated overnight at 4 °C in the dark in the bleaching solution (methanol and 6% H_2_O_2_) and then rehydrated in the opposite series of methanol/PBS. Then, the samples were blocked and permeabilized in 1X PBS-0.2% gelatin-0.5% Triton (PBSGT) for 48 hours. We next incubated the samples with primary antibodies against peripherin (1:500 dilution, #AB1530, Merck Millipore, Massachusetts, USA) and ChAT (1:100 dilution, #AB144P, Merck Millipore, Massachusetts, USA) diluted in PBSGT with 0.1% saponin for 14 days at 37 °C with mild agitation. After 14 days, the samples were rinsed 6 times over an entire day and incubated with anti-rabbit IgG Alexa Fluor^®^ 647 (A-21443, Molecular Probes, Oregon, USA) and anti-goat IgG Alexa Fluor^®^ 555 (A-21432, Molecular Probes, Oregon, USA). Secondary antibodies solution was filtered through a 0.22 µm syringe filter and incubated for 48 hours in PBSGT with 0.1% saponin at 37 °C with mild agitation. The samples were then rinsed 6 times with PBS over an entire day. Then, we performed the 3DISCO clearing protocol as previously described^12,13^. Briefly, the samples were incubated in the dark at room temperature in different concentrations of tetrahydrofuran (THF) diluted in water for 1.5 hours each (50% of THF, 80% of THF and two incubation in 100% THF) and then in dichloromethane (DCM) for 30 minutes followed by dibenzyl ether (DBE) for at least three hours. Images were acquired with an UltraMicroscope I (LaVision BioTec-Miltenyi, Bielefeld, Germany) assisted by Imspector Pro software (LaVision BioTec-Miltenyi Bielefeld, Germany). The light sheet created by two cylindrical lenses was generated by a laser with 2 specific wavelengths: 561 nm and 640 nm (Coherent Sapphire Laser, LaVision BioTec-Miltenyi). A protective dipping cap with a corrective lens was used for all acquisitions, and a binocular stereomicroscope (MXV10, Olympus) with a 2x objective (MVPLAPO, Olympus) was used. Samples were placed in a quartz tank (LaVision BioTec-Miltenyi) filled with DBE, and the illumination came from both sides. A Zyla SCMOS camera (2,048 × 2,048 pixels, Andor, Oxford Instruments) was used to acquire images. The step size between each image was fixed at 2 μm (NA = 0.5, 150 ms exposure). All images are generated as 16-bit.tiff files.

3D reconstructions are generated using the ImarisFileConverter (version 9.1.2, Bitplane) as Imaris Files (.ims). For 3D visualization on Imaris software (version 9.1.2, Bitplane), we used “volume”. We altered the normal shading view to obtain opaque visualizations. Optical slices are generated using the “orthoslicer” tool. 3D pictures and movies were generated using the “snapshot” and “animation” tools. Movie reconstructions with.tiff files were generated with ImageJ (1.50e, Java 1.8.0_60, 64-bit). We added titles and transitions with iMovie (version 10.1.1).

## Supplementary information

Supplementary file 1.

Supplementary file 2.

Supplementary Information file.
