# Supplementary material for: Clearing method for 3-dimensional immunofluorescence of osteoarthritic subchondral human bone reveals peripheral cholinergic nerves
Source: Sci Rep. 2020 Jun 1;10:8852. doi: 10.1038/s41598-020-65873-6 (PMC7264130; doi:10.1038/s41598-020-65873-6)
Supplement: Supplementary file 3 — Supplementary Information file. [file 41598_2020_65873_MOESM3_ESM.docx]

Supplementary Information file

**Clearing method for 3-dimensional immunofluorescence of osteoarthritic subchondral human bone reveals peripheral cholinergic nerves**

Alice Courties^1,2,3^, Morgane Belle^1,4^, Simge Senay^1,2^, Adeline Cambon-Binder^1,5^, Alain Sautet^1,5^, Alain Chédotal^1,4^, Francis Berenbaum*^1,2,3^, Jérémie Sellam^1,2,3^.

1. Sorbonne Université, Paris, France.

2. INSERM UMRS_938, CRSA, Paris, France.

3. Department of Rheumatology, Assistance Publique - Hôpitaux de Paris (AP-HP), Saint-Antoine Hospital, Paris, France.

4. INSERM, CNRS, Institut de la Vision, Paris, France.

5. Department of Orthopedic Surgery, AP-HP, Saint-Antoine Hospital, Paris, France.

**Supplementary file 1 : Peripheral nerves in OA human subchondral bone.** Immunofluorescence of peripheral nerves using peripherin (Prph) labelling in white.

**Supplementary file 2: Cholinergic nerves in OA human subchondral bone.** Co-Immunofluorescence of peripheral nerves marked by peripherin (Prph) in white and Choline acetyltransferase (ChAT) in red.

**
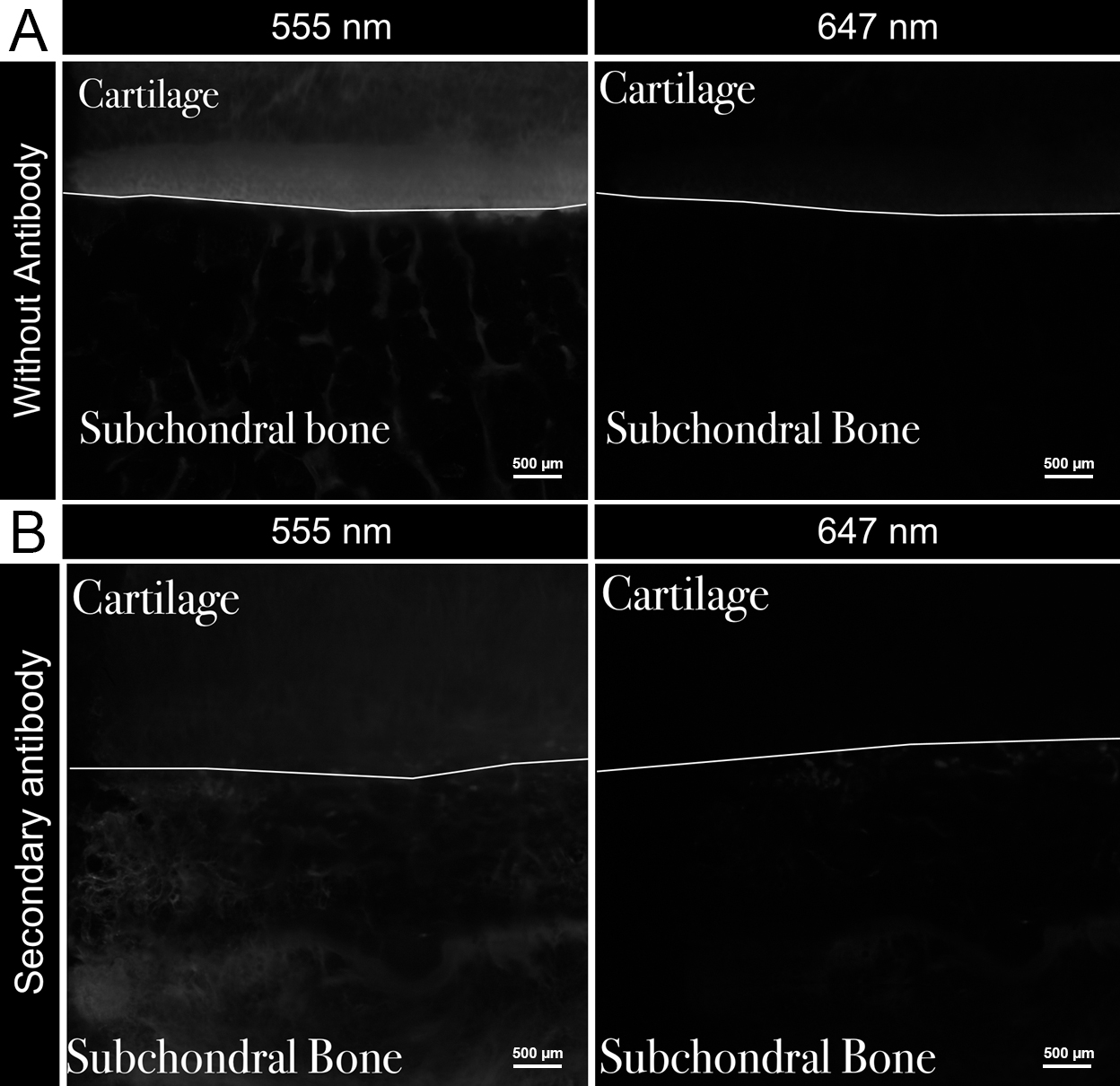
**

**Supplementary file 3: Figure A: Light-sheet microscopy analysis of cartilage and subchondral human OA plug without primary or secondary antibody.** The plug was incubated with the buffer containing phosphate buffer saline, gelatin and triton only. Two wavelengths were analysed at 555 and 647 nm using the same grey scale as for the analysis of the samples incubated with antibodies.

**Figure B: Light-sheet microscopy analysis of a cartilage and subchondral human OA plug with the secondary antibody only.** The plug was incubated with the buffer containing phosphate buffer saline, gelatin and triton first and then with secondary antibody anti-rabbit IgG Alexa Fluor^®^ 647 (A-21443, Molecular Probes, Oregon, USA) and anti-goat IgG Alexa Fluor^®^ 555 (A-21432, Molecular Probes, Oregon, USA) at dilution 1:500 dilution.
